# Draft Genome Sequence of the Wine Yeast Strain *Saccharomyces cerevisiae* I-328

**DOI:** 10.1128/genomeA.01520-17

**Published:** 2018-02-01

**Authors:** Andrey V. Mardanov, Alexey V. Beletsky, Mikhail A. Eldarov, Tatiana N. Tanashchuk, Svetlana A. Kishkovskaya, Nikolai V. Ravin

**Affiliations:** a Institute of Bioengineering, Research Center of Biotechnology of the Russian Academy of Sciences, Moscow, Russia; b Research Institute of Viticulture and Winemaking “Magarach” of the Russian Academy of Sciences, Yalta, Russia

## Abstract

*Saccharomyces cerevisiae* I-328 is a yeast strain used for production of sherry-like wine in Russia. Here we report the draft genome sequence of this strain, which will facilitate comparative genomic studies of yeast strains used for winemaking.

## GENOME ANNOUNCEMENT

Biological maturation is a traditional wine-making process performed in the course of aerobic respiration by a special group of *Saccharomyces cerevisiae* wine strains called flor yeasts (1). Flor yeasts are able to float on the surface of the wine and form a biofilm in which they oxidize ethanol, with the formation of specific aromatic and flavor compounds that impart a specific taste to the wine. This methodology is used in the production of sherry-like wines around the world, mostly in Spain and France. Comparative genomics studies of the wine yeast strains have revealed genetic features specific for flor yeasts (2, 3).

Sherry-like wines have been produced for a long time in Russia and some countries of Eastern Europe. The Magarach Collection of the Microorganisms for Winemaking contains a large number of *S. cerevisiae* wine strains isolated from natural sources and obtained from other collections. Wine strain I-328, obtained from the Institute of the Fermentation Industry (Berlin, Germany) in 1948, was similar to flor yeast strains in some physiological characteristics and able to form a flor in the process of sherry wine aging (4).

In this work, the genome sequence of *S. cerevisiae* I-328 was obtained by using Illumina HiSeq2500 technology. Isolation of the genomic DNA of strain I-328 was carried out from a culture grown from a separate colony in a yeast extract-peptone-dextrose (YEPD) medium at 20°C. The sequencing of a TruSeq DNA library (250-nucleotide [nt] reads) generated 15,314,404 reads. Sequencing primers were removed using Cutadapt (5) and low-quality read regions were trimmed using Sickle (https://github.com/najoshi/sickle). Illumina reads were *de novo* assembled using SPAdes 3.7.1 (6). Contigs shorter than 200 bp were discarded. The resulting nuclear genome assembly had a length of 11,611,250 bp divided into 561 contigs; the *N*_50_ contig length was 62,127 bp. At the ends of the three contigs, telomere repeats of *S. cerevisiae* [T(G)_2–3_ (TG)_1–6_] (7, 8) were found. The mitochondrial genome was assembled into a single contig of 81,994 bp. Protein-coding genes were predicted using Augustus 3.0.3 (9) trained on an *S. cerevisiae* S288C data set. A total of 5,341 and 11 protein-coding genes were predicted in the nuclear and mitochondrial genomes, respectively. By use of the tRNA-scanSE program (10), 273 tRNA genes were found in the nuclear genome. The search for tRNA in the mitochondrial genome was performed using the MFannot program organelle genome annotation Web server (http://megasun.bch.umontreal.ca/cgi-bin/mfannot/mfannotInterface.pl), and 23 tRNA genes were found. Annotation of protein-coding genes was performed using a BLASTP search against *S. cerevisiae* S288C proteins and a nonredundant protein sequence database.

Analysis of the genome of strain I-328 revealed the presence of an 11-nt deletion characteristic of flor yeast strains in the promoter of the adhesin FLO11 gene (11). However, a 24-nt deletion in the internal transcribed spacer 1 (ITS1) region found in Spanish sherry yeast strains and the C insertion characteristic of French Jura flor strains (12, 13) were absent in strain I-328. The data reported in this work will be useful for further research in the field of comparative genomics of wine and flor yeasts.

### Accession number(s).

This BioProject has been deposited in GenBank under number PRJNA414946. This genome sequence has been deposited at DDBJ/EMBL/GenBank under the accession number PEJR00000000.
